# Caffeic Acid Phenethyl Ester Protects Against Doxorubicin-Induced Cardiotoxicity via Inhibiting the ROS-MLKL-Mediated Cross-Talk Between Oxidative Stress and Necroptosis

**DOI:** 10.3390/biom15060783

**Published:** 2025-05-28

**Authors:** Chenying Jiang, Tinghuang Zhang, Jiawen Gu, Chenjun Shen, Hang Gao, Hai An, Chen Wang, Jiahui Lu, Shengzhang Lin, Huajun Zhao, Zhihui Zhu

**Affiliations:** 1 School of Pharmaceutical Sciences, Zhejiang Chinese Medical University, Hangzhou 311402, China; 2 Academy of Chinese Medical Sciences, Zhejiang Chinese Medical University, Binwen Rd., Hangzhou 310053, China; 3 School of Medicine, Hangzhou City University, Hangzhou 310015, China

**Keywords:** caffeic acid phenethyl ester, Doxorubicin, cardiotoxicity, ROS, necroptosis, cross-talk

## Abstract

Purpose: Doxorubicin (DOX) is a broad-spectrum anti-tumor anthracycline drug. However, its clinical application is greatly limited due to the side effect of cardiotoxicity. Caffeic acid phenethyl ester (CAPE) is one of the major biologically active compounds isolated from propolis, which is effective in the treatment of cardiovascular diseases. The purpose of this study aimed to explore the possible mechanism of CAPE’s protective effect on DOX-induced cardiotoxicity (DIC). Methods: In vivo, a DIC model was established by the intraperitoneal injection of 3 mg/kg DOX. The cardiac function of mice was monitored by electrocardiograms. Histopathological changes in myocardial tissue were detected by H&E staining. Serum samples were tested for the level of markers of myocardial injury. In vitro, transmission electron microscopy was used to assess the mitochondrial damage. Oxidative stress was measured by flow cytometry and mitochondrial respiration analysis. Necroptosis pathway changes were detected by Western blotting. Furthermore, the overexpression plasmid of a key necroptosis gene, necroptosis inhibitor or ROS inducer/inhibitor was applied to H9c2 and AC16 cells to explore whether CAPE exerted a protective effect against DIC through the cross-talk mediated by ROS and MLKL. Results: CAPE could improve the cardiac function and protect against myocardial tissue. CAPE pre-administration treatment attenuated the DOX-induced generation of ROS, protected mitochondrial functions and inhibited necroptosis. Moreover, there was cross-talk between the ROS and necroptosis. CAPE could protect against DIC by inhibiting the ROS-MLKL signaling that regulated the cross-talk. Conclusions: CAPE alleviated the oxidative stress and necroptosis of DIC, indicating that the cross-talk mediated by ROS-MLKL signaling may be a potential therapeutic mechanism for clinical DIC.

## 1. Introduction

Doxorubicin (DOX) is a broad-spectrum anthracycline, which is a foundation of chemotherapy regimens for some malignant tumors, including malignant lymphoma, breast cancer, bronchopulmonary cancer and ovarian cancer [1]. However, the clinical application of DOX is limited by its dose-dependent and irreversible cardiotoxicity, which further develops into congestive heart failure [2]. The relationship between congestive heart failure and the cumulative dose of DOX has been fully proved [3]. Dexrazoxane (Dex) is currently the only cardioprotective agent approved by the U.S. Food and Drug Administration (FDA) to prevent DOX-induced cardiotoxicity (DIC) in breast cancer treatments [4]. But it may aggravate bone marrow suppression and lead to secondary malignant tumors after long-term use [5,6]. Thus, the exploration of safe and effective drugs against DIC is urgent.

**Figure 1:**
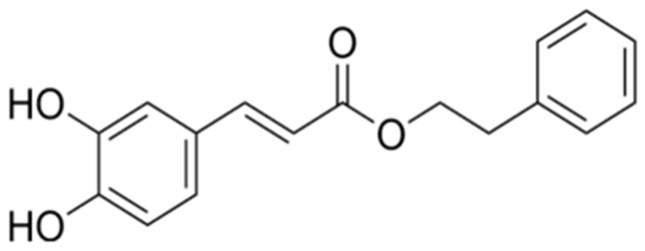
The chemical formula of CAPE.

Natural products play an important role in the process of drug discovery. Caffeic acid phenethyl ester (CAPE) is one of the content-determination components of propolis [7], which possesses a wide range of biological activities, such as anti-cancer, antioxidant, anti-inflammatory and immunoregulation (Figure 1) [8,9,10]. The remarkable antioxidant activity of CAPE is widely acknowledged. CAPE is a hydroxyl derivative of cinnamic acid. Compared with other phenolic acids, the existence of the CH_2_=CH-COOH group in cinnamic acid has achieved stronger antioxidant activity [11]. At present, the antioxidant activity and cytoprotective activity of CAPE against ischemia–reperfusion injury in various target tissues, such as the heart, skeletal muscle, intestine, testicle and lung, have been confirmed [12,13,14,15,16]. Moreover, several studies have demonstrated that CAPE could enhance the sensitivity of DOX in cancer therapy, indicating that CAPE holds great value in the adjuvant treatment of cancer with DOX [17,18]. However, the potential role and mechanism of CAPE in DIC need to be further studied.

The mechanisms of DIC are not fully clear. Several potential etiological factors have been classically considered to induce DIC, including oxidative stress, apoptosis, autophagy, mitochondrial dysfunction, the accumulation of free iron, the disruption of Ca^2+^ homeostasis and DNA damage [19]. Oxidative stress is a main cause of DIC according to the present literature [20,21,22]. Mitochondria are the most important organelles for oxidative stress. In mitochondria, DOX can be reduced by mitochondrial complex I or cytochrome P450 reductase to produce semiquinone radicals that react with oxygen molecules and re-oxidize into parent DOX molecules during the redox cycle [23]. This process produces different oxidative stress products, which can be further converted into highly reactive and toxic hydroxyl radicals by Fenton and Haber–Weiss reactions [24]. However, the low content of antioxidant enzymes in cardiomyocytes makes cardiomyocytes more sensitive to oxidative stress. Therefore, the high level of ROS produced by DOX leads to severe myocardial cell injury [25].

As a key factor of DIC, necroptosis is a type of programmed necrosis cell death, which is characterized by cell swelling and the loss of plasma membrane integrity and accompanied by the release of damage-related molecular patterns and the occurrence of inflammation [26]. After a DOX treatment, the death receptor was significantly increased at the gene and protein levels, especially tumor necrosis factor receptor 1 (TNFR1) [27,28]. The activation of TNFR1 can stimulate receptor-interacting serine/threonine-protein kinase-1 (RIPK1) to further recruit receptor-interacting serine/threonine-protein kinase-3 (RIPK3), leading to the formation of necrosomes. In addition, necrosomes promote mixed-lineage kinase domain-like (MLKL) phosphorylation, which is translocated to the plasma membrane after forming a polymer. The MLKL polymer eventually leads to plasma membrane rupture and the release of inflammatory factors [29].

The relationship between ROS and necroptosis has been reported [30,31,32]. A large amount of mitochondrial ROS (Mito-ROS) can induce the binding of pore-forming protein Gasdermin D (GSDMD) to the mitochondrial membrane , and then promote the transformation to RIPK1/RIPK3/MLKL-dependent necroptosis [33,34]. In addition, necroptosis can also reversely promote mitochondrial dysfunction, triggering intracellular oxidative stress [35]. However, this cross-talk between oxidative stress and necroptosis has not been reported on regarding the mechanism of DIC. The purpose of this study was to verify the cross-talk mechanism between oxidative stress and necroptosis in DIC, and by which CAPE coordinated the cross-talk via inhibiting ROS-MLKL signaling to protect against DIC, so as to provide an alternative cardio-protectant for DIC.

## 2. Materials and Methods

### 2.1. Reagents and Chemicals

CAPE (#HY-N0274, purity > 98%) and DOX (#HY-15142) were purchased from MedChemExpress (Monmouth Junction, NJ, USA). The assay kits for cardiac troponin-I (cTn-I), creatine kinase-MB (CK-MB) and lactate dehydrogenase (LDH) were purchased from the Meimian company (Yancheng, China). The hematoxylin and eosin (H&E) kit (#C0105S), ROS assay kit (#S0033S) and MitoSOX Red assay kit (#S0061S) were purchased from Beyotime Biotechnology (Shanghai, China). The glutathione content assay kit (#BC1175) was purchased from Solarbio (Beijing, China). The cell energy metabolism analysis kit was purchased from Huawei Zhongyi (Beijing, China). Anti-RIPK3 (#ER1901-27) was obtained from HUABIO (Beijing, China). Anti-RIPK1 (ab300617) and MLKL (ab243142) were obtained from Abcam (Cambridge, UK). Phospho-MLKL (Ser358) polyclonal antibody (#PA5-105678) was obtained from Thermo Fisher Scientific (Waltham, MA, USA). 3-(4,5-dimethyl-2-thiazolyl)−2,5-diphenyl-2Htetrazolium bromide (MTT), dimethyl sulfoxide (DMSO) and hydrogen peroxide (H_2_O_2_) were purchased from Sigma-Aldrich (St. Louis, MO, USA).

### 2.2. Cell Lines and Culture

Rat cardiomyoblast cells (H9c2) and human myocardial cells (AC16) were purchased from the National Collection of Authenticated Cell Cultures (Shanghai, China). All cells were cultured in DMEM/F12 medium supplemented with 10% (*v/v*) fetal bovine serum, 100 U/mL penicillin and 100 μg/mL streptomycin at 37 °C with 5% CO_2_.

### 2.3. Animals and Treatments

All animal experiments were conducted in accordance with the animal experiment guidelines of Zhejiang Chinese Medical University Laboratory Animal Research Center (laboratory animal license number: SYXK (Zhejiang) 2021-0012). Approval for animal use was granted by the Animal Ethical and Welfare Committee of Zhejiang Chinese Medical University (approval no.: IACUC-202312-05).

Ninety male BALB/c mice (23 ± 2 g, 6–8 weeks old) were purchased from Hangzhou Qizhen Experimental Animal Technology Co., Ltd. (Hangzhou, China) and kept in a specific pathogen-free (SPF) barrier system. The mice were acclimatized for at least 1 week prior to the experiment. They were housed in ventilated cages with corncob bedding (5 mice per cage) under controlled environmental temperature conditions (22 ± 2 °C) in a 12 h light/dark cycle. The animals were maintained on standard food pellets and tap water ad libitum.

After acclimation for one week, ninety mice were randomly divided into nine groups (*n* = 10): control group, DOX group, CAPE (10 mg/kg) group, CAPE (20 mg/kg) group, CAPE (40 mg/kg) group, DOX + CAPE (10 mg/kg) group, DOX + CAPE (20 mg/kg) group, DOX + CAPE (40 mg/kg) group and DOX + positive control drug (dexrazoxane, Dex) (90 mg/kg) group. The CAPE monotherapy groups (10/20/40 mg/kg) received daily intraperitoneal injections of CAPE, while the remaining six groups were administered an equivalent volume of blank solvent via intraperitoneal injection for 21 days. Starting on day 7, the model group (DOX group) and DOX combination groups were injected intraperitoneally with DOX (3 mg/kg) every 2 days for 14 days (a total of 7 times), which resulted in a cumulative DOX dose of 21 mg/kg to establish the DIC model. The other four groups received equivalent volumes of saline. In the combination groups, CAPE and Dex were administered via an intraperitoneal injection 30 min prior to the DOX administration. The body weight was measured and recorded daily using an electronic animal scale. On day 21, the mice were anesthetized with isoflurane (4% induction, 1.5–2% maintenance in 100% oxygen), and surface electrocardiograms were recorded using an MP160 physiological recording system. And blood samples and the hearts of the mice were collected. The heart samples were fixed in formalin, while the serum samples were frozen at −80 °C in an ultra-low temperature freezer for subsequent experiments.

### 2.4. Cell Viability Assay

Cells (3 × 10^3^ cells per well) were seeded in a 96-well plate and cultured overnight. The cells were then treated with different concentrations of CAPE (0, 5, 10, 20, 40, 80 µg/mL). After incubation with CAPE for 6 h, the cells were co-treated with 2.5 µM DOX for another 18 h. An oxidative stress model was induced by incubation with 400 μM H_2_O_2_ for 6 h. To assess the influence of ROS and necroptosis inhibitors on the inhibition of cardiomyocytes by DOX, the cells were seeded into plates as mentioned above, followed by the incubation of N-acetylcysteine (NAC) (200 µM) or Necrostatin-1 (Nec-1) (100 µM) for 6 h, and DOX (2.5 µM) for another 18 h. After 24 h, the cells were incubated in 20 μL of MTT solution (5 mg/mL) for 4 h at 37 °C to form violet formazan crystals. Subsequently, DMSO was added into each well to dissolve the violet formazan crystals. Then, the relative cell viability was measured by a Multi-Mode Reader (BioTek, Winooski, VT, USA) using a 570 nm filter.

### 2.5. Surface Electrocardiogram (ECG) Assessment of Cardiac Function

The mice were lightly anesthetized with 1.5% isoflurane in oxygen. Then, they were placed in a supine position and kept warm at 37 °C on a heating pad. The limbs of the mice were attached to an MP160 polygraph system (BIOPAC, Goleta, CA, USA) to record the ECG.

### 2.6. Histopathologic Assay

The heart samples were soaked in 10% buffered formalin for at least 24 h and then embedded in paraffin. The embedded sections were cut with a 5 μm thickness, and then dewaxed and dehydrated after drying. H&E staining was performed according to the manufacturer’s instructions to evaluate the heart toxicity.

### 2.7. Measurement of Serum Levels of cTn-I, CK-MB and LDH

The serum levels of cTn-I, CK-MB and LDH were measured according to the manufacturer’s instructions for each kit.

### 2.8. Transmission Electron Microscopy (TEM)

The integrity and the morphological change of cells were observed by TEM. The samples were fixed by pre-cooled 2.5% glutaraldehyde and sent to the Medical Research Centre of Zhejiang Chinese Medical University for further processing and slicing. Images were acquired under a transmission electron microscope (HITACHI, Hitachi, Japan).

### 2.9. Oxidative Stress Detection

The collected cells were stained with a blank medium containing Dichlorodihydrofluorescein Diacetate (DCFH-DA) (1 µM) at 37 °C for 20 min. Subsequently, the cells were washed and harvested. The ROS level was evaluated by a CytoFlex flow cytometer (Beckman, Brea, CA, USA). A blank medium that contained MitoSOX Red (5 µM) was incubated to detect the level of mitochondrial peroxidation by using the same method. The level of glutathione (GSH) was measured according to the manufacturer’s instructions for the kit.

### 2.10. Mitochondrial Respiration Analysis

The mitochondrial oxygen consumption rate (OCR) was detected by a Cell Energy Metabolism Analysis System (Oroboros, Innsbruck, Austria). First, the cell suspension and respiratory media were added to the cabin. Subsequently, the following compounds were injected: 1 μM oligomycin (Omy), 5 μM carbonyl cyanide-4-(trifluoromethoxy) phenylhydrazone (FCCP) and a mixture of 1 μM rotenone (Rot) plus 1 μM antimycin A (Ama) to determine the basal respiration, ATP production, maximal respiration and spare respiratory capacity.

### 2.11. Western Blotting

The collected cells were lysed with a radio immunoprecipitation assay buffer supplemented with phenylmethylsulfonyl fluoride. Equivalent amounts of protein were separated by SDS-PAGE and transferred to polyvinylidene difluoride membranes (Millipore, Birica, MA, USA). The membranes were blocked with 5% bovine serum albumin and incubated with the specified primary antibodies overnight at 4 °C, followed by incubation with the corresponding secondary antibodies. The expression of the target proteins was finally detected using enhanced chemiluminescence (Bio-Rad, Hercules, CA, USA) for chemiluminescence detection.

### 2.12. Immunohistochemistry Assay (IHC)

The approach used for the section preparation is described in the Materials and Methods Section “Histopathologic assay”. A 3% hydrogen peroxide solution was first used to block the endogenous peroxidase, followed by antigen repair with sodium citrate buffer. The sections were incubated with primary antibodies overnight at 4 °C. The next day, the sections were incubated in the presence of a horseradish peroxidase-conjugated secondary antibody, stained with 3,3′-diaminobenzidine and counterstained with hematoxylin. Finally, the slides were observed by bright-field microscopy (ZEISS, Jena, Germany).

### 2.13. Plasmid Transfection

The GV657 lentiviral vector expressing two different overexpressed plasmids that target rat MLKL and human MLKL or a negative control plasmid were purchased from Genechem (Shanghai, China). The cells were seeded into 6-well plates and incubated until they reached 70% confluence. The transfection complex was mixed with 2 µg of plasmid DNA and 2 µL of Lipofectamine 2000 (Invitrogen), incubated for 20 min at room temperature and then added to the cells. After 6 h, the complete medium was added into the cells and cultured for 48 h. The MLKL-overexpressing cells were evaluated using Western blotting analysis.

### 2.14. Statistical Analysis

All the analyses were performed using GraphPad Prism 5.0 software (San Diego, CA, USA). The studies were designed to generate groups of equal size using randomization and blinded analysis. All the experiments were independently repeated at least *n* = 3 times with similar results. The measurement data are expressed as the mean ± standard deviation (SD). For comparisons between two groups, Student’s *t*-tests were applied. For multi-group comparisons, one-way ANOVA was used if the data met the assumptions of normality and homogeneity of variance. When the ANOVA F statistic reached statistical significance (*p* < 0.05), the post hoc test of Tukey’s test was performed for pairwise comparisons. Variables that did not meet normal distribution or homogeneity criteria were analyzed using Games–Howell’s or Dunnett’s tests. Differences were considered statistically significant according to the threshold of *p* < 0.05.

## 3. Results

### 3.1. CAPE Alleviated DOX-Induced Myocardial Injury In Vivo

**Figure 2:**
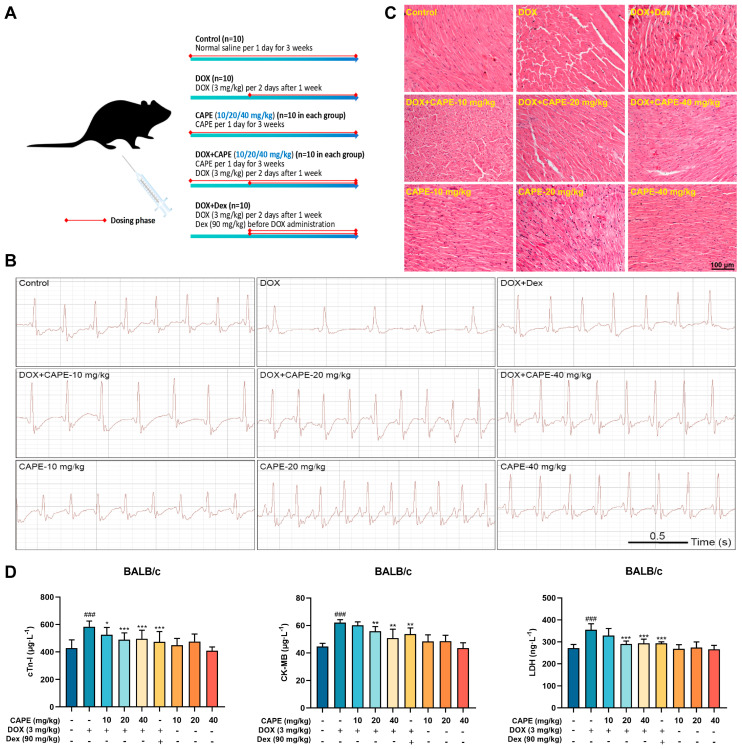
CAPE alleviated the DOX-induced myocardial injury in vivo. (**A**) Schematic diagram of animal experiment process. (**B**) ECG was used to evaluate the cardiac function of the mice in each group. (**C**) H&E staining was used to detect the cardiac toxicity. Scale bar: 100 μm. (**D**) The ELISA kits were used to detect the levels of cTn-I, CK-MB and LDH in serum. Values are represented as the $\bar{x}$ ± SD (*n* = 10). *^###^ p* < 0.001 vs. control; * *p* < 0.05, ** *p* < 0.01, *** *p* < 0.001 vs. DOX-treatment group.

To confirm the protective effects of CAPE on DIC, the mice were subjected to the treatments as indicated in the schematic plan (Figure 2A). The DIC mice were observed to have significant cardiotoxicity after the treatment with DOX at a cumulative dose of 21 mg/kg. The administration methods of Dex referred to clinical practice. The cardiac functions were monitored by a surface ECG (Figure 2B). The results show that the abnormal ECG changes, such as the slow heart rate, prolonged QRS duration and reduction in QRS amplitude, were observed in the DOX group compared with the control group, and CAPE could effectively relieve such abnormal changes (Figure 2B). The H&E staining exhibited that DOX could increase the intercellular spaces and the disturbance of the nuclear arrangement. CAPE significantly protected against cardiomyocyte damage caused by DOX (Figure 2C). In addition, DOX elevated the levels of cTn-I, CK-MB and LDH, while CAPE could reduce their levels (Figure 2D).

### 3.2. CAPE Alleviated DOX-Induced Myocardial Injury In Vitro

**Figure 3:**
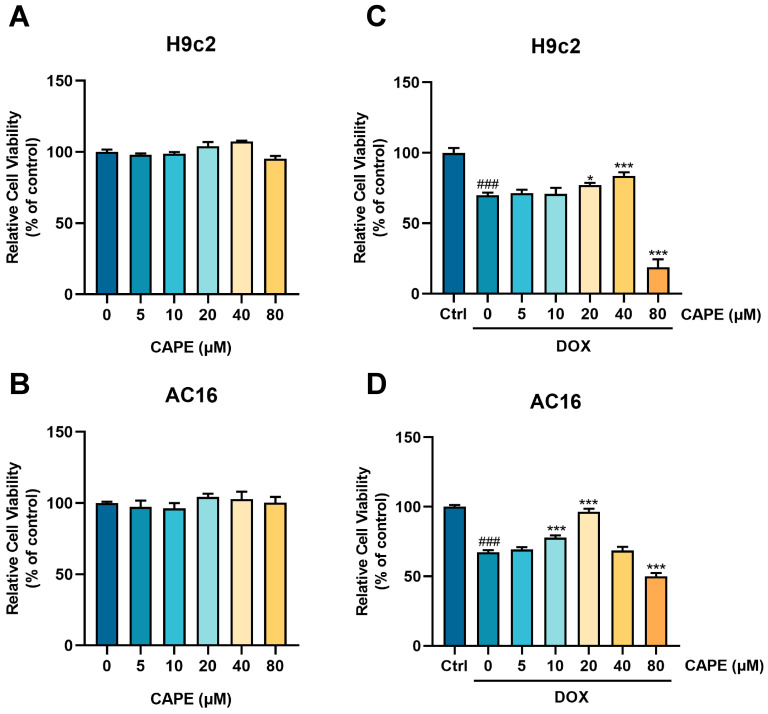
CAPE alleviated the DOX-induced myocardial injury in vitro. The (**A**) H9c2 and (**B**) AC16 cells were incubated with CAPE (0–80 µM) for 24 h. The cell viability was examined by MTT assays. (**C**) H9c2 cells and (**D**) AC16 cells were pre-treated with CAPE (0–80 µM) for 6 h, followed by 18 h of incubation with DOX (2.5 µM). The cell viability was assessed by MTT assays. Values are represented as the $\bar{x}$ ± SD. *^###^ p* < 0.001 vs. control; * *p* < 0.05, *** *p* < 0.001 vs. DOX treatment group.

H9c2 and AC16 cells were subjected to the treatment of 2.5 μM DOX to establish a DIC model in vitro to further evaluate the cardioprotective potential of CAPE. The concentration of DOX was based on our previous studies that showed significant toxicity [36]. After 24 h of treatment, CAPE was safe for the H9c2 and AC16 cells in the concentration range of 5–80 μM (Figure 3A,B). The cells were pre-treated with CAPE for 6 h and then treated with DOX for another 18 h. After 24 h of incubation, DOX reduced the cell viability, while CAPE significantly reversed the cell viability (Figure 3C,D). Working concentrations of 20 and 40 μM of CAPE were chosen for the H9c2 cells and 10 and 20 μM for the AC16 cells.

### 3.3. CAPE Attenuated DOX-Induced Oxidative Stress

**Figure 4:**
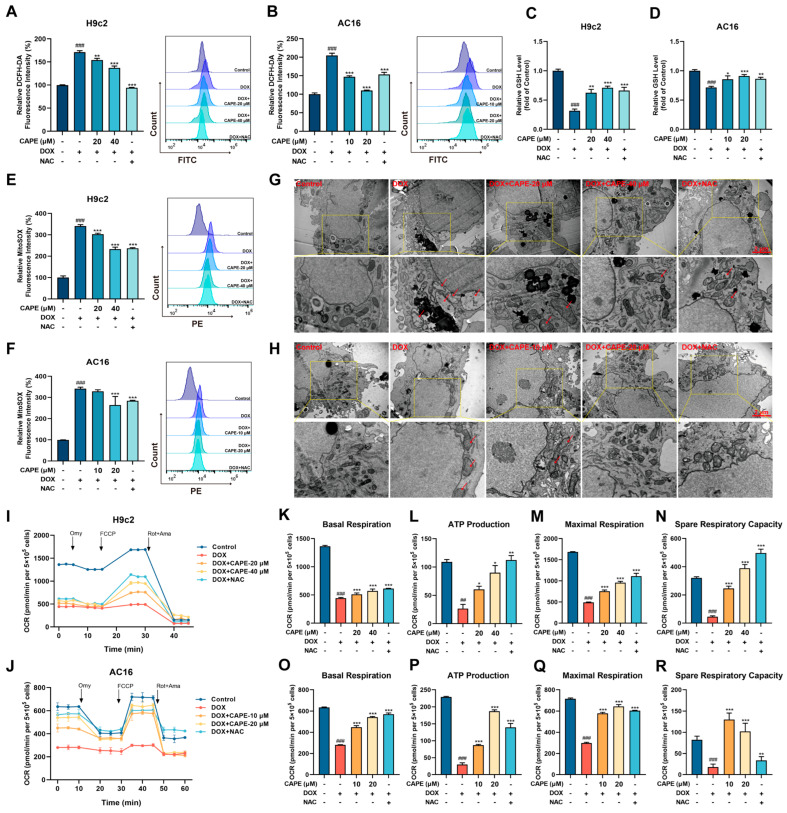
CAPE attenuated the DOX-induced oxidative stress. The H9c2 and AC16 cells were pre-treated with CAPE or NAC for 6 h, followed by 18 h of incubation with DOX (2.5 μM). The ROS levels of H9c2 (**A**) and AC16 (**B**) were assessed and quantified by flow cytometry after the DCFH-DA staining. A GSH kit was used to assess the GSH levels of the H9c2 (**C**) and AC16 (**D**). The Mito-ROS levels of the H9c2 (**E**) and AC16 (**F**) were assessed and quantified by flow cytometry after the MitoSOX Red staining. The mitochondrial damage of the H9c2 (**G**) and AC16 (**H**) were shown by TEM. Scale bar: 1 μm. Red arrows indicate damaged mitochondria. The OCRs of the H9c2 (**I**) and AC16 (**J**) were examined by an O2K cell energy metabolism analysis system. The basal respiration, ATP production, maximal respiration and spare respiratory capacity of the H9c2 (**K**–**N**) and AC16 (**O**–**R**) were quantified. Values are represented as the $\bar{x}$ ± SD. *^##^ p* < 0.01, *^###^ p* < 0.001 vs. control; * *p* < 0.05, ** *p* < 0.01, *** *p* < 0.001 vs. DOX treatment group.

Oxidative stress is considered to be the critical inducement of DIC. NAC, a ROS inhibitor with a thiol group, was found to confer strongly antioxidant activity. We used NAC as a positive drug to verify the antioxidant capacity of CAPE in the DOX-induced cells. To evaluate the effect of CAPE against oxidative stress, the ROS generation and GSH content were examined in the H9c2 and AC16 cells. Compared with the control group, the ROS level in the cardiomyocytes was significantly increased and the GSH level decreased in the DOX group (Figure 4A,B). After the CAPE pre-treatments, the ROS levels were then decreased (Figure 4A,B). In addition, CAPE upregulated the levels of GSH (Figure 4C,D). Excessive ROS further attack mitochondria, leading to oxidative stress damage. The levels of Mito-ROS in the cardiomyocytes were examined by flow cytometry. CAPE could significantly reduce the DOX-induced considerable increase in Mito-ROS (Figure 4E,F). Since the mitochondrion is the main organelle for ROS production, the effects of CAPE on DOX-induced mitochondrial damage and dysfunction were further investigated. First, the TEM images show, as the red arrow refers to, the DOX-induced mitochondrial damage in the cardiomyocytes, which was characterized by mitochondrial swelling, broken and reduction in cristae, and the occurrence of mitochondrial vacuolization (Figure 4G,H). After the CAPE pre-treatments, the mitochondrial damage was alleviated (Figure 4G,H). The mitochondrial respiration potency of the H9c2 and AC16 cells treated with DOX with or without CAPE pre-treatment was also measured. DOX induced a significant reduction in the mitochondrial respiration potency, while the pre-treatment with CAPE alleviated the DOX-induced inhibition of mitochondrial respiration in the H9c2 and AC16 cells (Figure 4I,J). The mitochondrial respiration parameters, such as the basal respiration, ATP production, maximal respiration and spare respiratory capacity, were also ameliorated after the CAPE pre-treatments (Figure 4K,R). Our results indicate that CAPE had similar effects to NAC, which attenuated the DOX-induced mitochondria-derived oxidative stress.

### 3.4. CAPE Inhibited DOX-Induced Necroptosis by Inhibiting the RIPK1/RIPK3/MLKL Pathway

**Figure 5:**
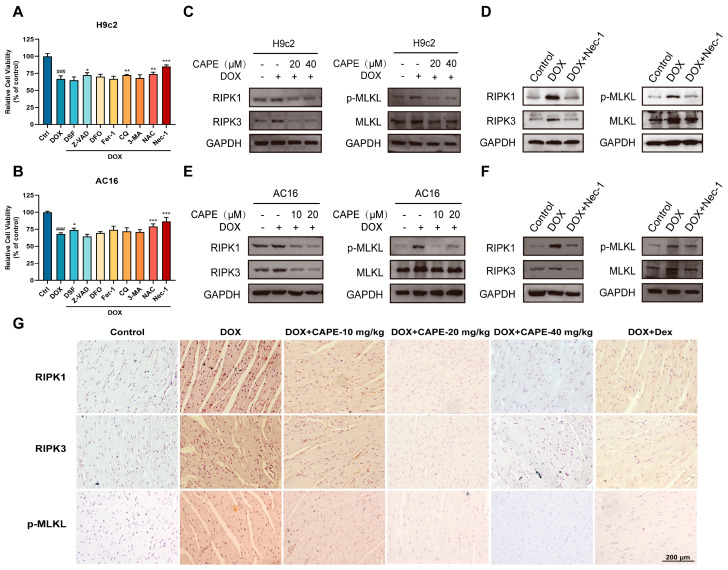
CAPE inhibited the DOX-induced necroptosis by inhibiting the RIPK1/RIPK3/MLKL pathway. The H9c2 (**A**) and AC16 (**B**) cells were pre-treated with various death inhibitors, namely, DSF (10 μM), Z-VAD (10 μM), DFO (10 μM), Fer-1 (10 μM), CQ (10 μM), 3-MA (10 μM), NAC (200 μM) and Nec-1 (100 μM) for 6 h, followed by a treatment with DOX for a total of 24 h. The cell viability was examined by MTT assays. The H9c2 (**C**,**D**) and AC16 (**E**,**F**) cells were pre-treated with CAPE or Nec-1 for 6 h, followed by 18 h of incubation with DOX (2.5 μM). The protein levels related to the RIPK1/RIPK3/MLKL pathway were examined by Western blotting. (**G**) The expression levels of RIPK1, RIPK3 and p-MLKL in the mouse hearts were examined by IHC. Scale bar: 100 μm. Values are represented as the $\bar{x}$ ± SD. *^###^ p* < 0.001 vs. control; * *p* < 0.05, ** *p* < 0.01, *** *p* < 0.001 vs. DOX treatment group.

Nec-1 is a necroptosis inhibitor that targets RIPK1. Among various death inhibitors, the downregulation of cell viability by DOX (2.5 μM) was rescued most significantly by Nec-1 (Figure 5A,B), which suggests that necroptosis may play a more important role in DIC. We used Nec-1 as a positive drug to prove the effects of CAPE on DOX-induced necroptosis. The major protein expressions involved in necroptosis-related pathways were examined by Western blotting. Notably, p-MLKL was a crucial indicator to evaluate the occurrence of necroptosis. After the DOX treatment, the protein levels of RIPK1, RIPK3 and p-MLKL/MLKL related to the degree of necroptosis were significantly upregulated compared with the control group (Figure 5C,F). However, the increased protein levels could be inhibited after pre-treatments of CAPE or Nec-1 (Figure 5C,F). Meanwhile, immunohistochemistry staining results showed that the expressions of RIPK1, RIPK3 and p-MLKL were also decreased in DOX-induced necroptosis after the pre-treatments of CAPE (Figure 5G). The results indicate that CAPE had similar effects to Nec-1, which inhibited the DOX-induced necroptosis by inhibiting the RIPK1/RIPK3/MLKL pathway.

### 3.5. The Cross-Talk Between Oxidative Stress and Necroptosis Played a Vital Role in DOX-Induced Cardiotoxicity

**Figure 6:**
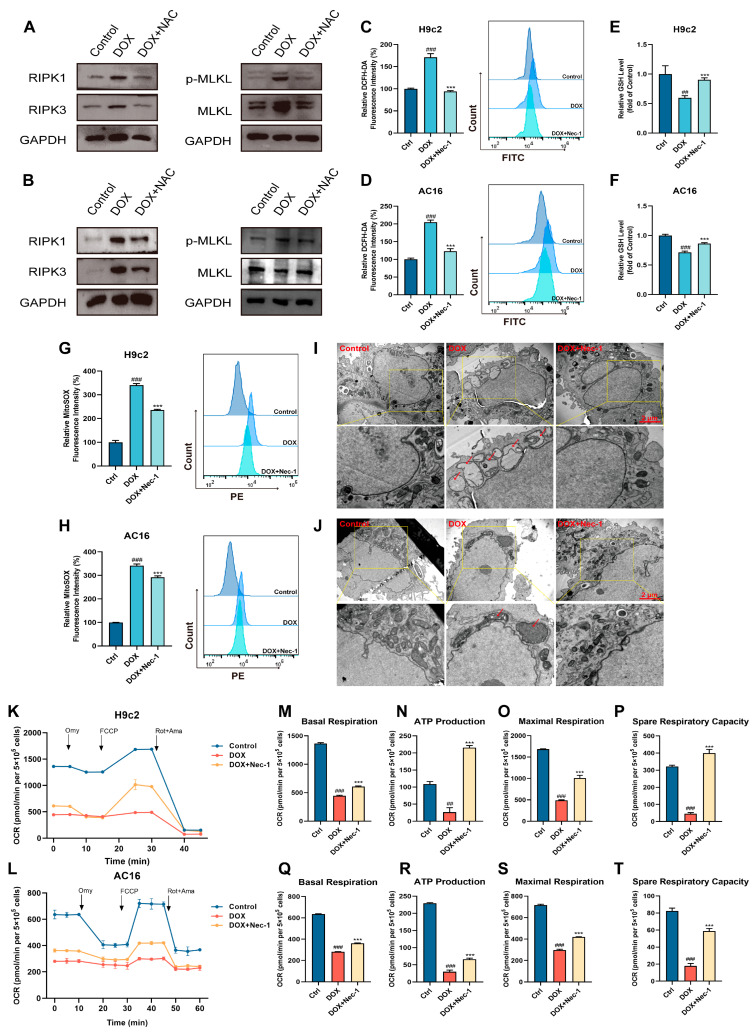
The cross-talk between oxidative stress and necroptosis played a vital role in the DOX-induced cardiotoxicity. The H9c2 and AC16 cells were pre-treated with NAC for 6 h, followed by 18 h of incubation with DOX (2.5 μM). The protein levels of the H9c2 (**A**) and AC16 (**B**) cells related to the RIPK1/RIPK3/MLKL pathway were examined by Western blotting. The H9c2 and AC16 cells were pre-treated with Nec-1 for 6 h, followed by 18 h of incubation with DOX (2.5 μM). (**C**,**D**) The ROS level was assessed and quantified by flow cytometry after the DCFH-DA staining. (**E**,**F**) The GSH levels were assessed. (**G**,**H**) The Mito-ROS level was assessed and quantified by flow cytometry after MitoSOX Red staining. The mitochondrial damage of H9c2 (**I**) and AC16 (**J**) cells were shown by TEM. Scale bar: 1 μm. (**K**,**L**) OCR was examined by an O2K cell energy metabolism analysis system. Basal respiration, ATP production, maximal respiration and spare respiratory capacity of H9c2 (**M**–**P**) and AC16 (**Q**–**T**) cells were quantified. Values are represented as the $\bar{x}$ ± SD. *^##^ p* < 0.01, *^###^ p* < 0.001 vs. control; *** *p* < 0.001 vs. DOX treatment group.

The above studies indicate the effects of CAPE that could inhibit oxidative stress and RIPK1/RIPK3/MLKL-dependent necroptosis in DIC. However, the cross-talk between oxidative stress and necroptosis in DIC has not been reported. Thus, to investigate the underlying relationships between oxidative stress and necroptosis under a DOX treatment, NAC was added to explore the role of DOX in induced necroptosis, and Nec-1 was added to study the role of DOX-induced oxidative stress in cardiomyocytes. We measured the expressions of key proteins in DOX-induced necroptosis activation, such as RIPK1, RIPK3 and p-MLKL/MLKL in the H9c2 and AC16 cells after NAC pre-administration. Robust elevations of RIPK1, RIPK3 and p-MLKL/MLKL protein levels were observed after exposure to DOX, and this upregulation could be significantly inhibited by NAC (Figure 6A,B). Furthermore, the increased levels of ROS induced by DOX were markedly attenuated by Nec-1 compared with the DOX group (Figure 6C,D). Similarly, Nec-1 could also prevent the DOX-induced downregulation of GSH (Figure 6E,F). Nec-1 could also significantly reduce the considerable DOX-induced increase in Mito-ROS (Figure 6G,H). In addition, the DOX-induced mitochondrial damage and dysfunction were equally investigated. The mitochondrial injury in the cardiomyocytes could be attenuated by Nec-1 compared with the DOX group (Figure 6I,J). Meanwhile, our results of mitochondrial respiration potency showed that the subdued mitochondrial respiration in the H9c2 and AC16 cells triggered by DOX, such as basal respiration, ATP production, maximal respiration and spare respiratory capacity, were obviously elevated after the Nec-1 pre-treatments **(**Figure 6K–T). Therefore, we could infer that there was an interactive loop between necroptosis and oxidative stress in the DOX-induced cardiomyocytes, which together promoted the occurrence of cardiotoxicity.

### 3.6. CAPE-Inhibited ROS-MLKL Signaling Mediated the Cross-Talk Between Oxidative Stress and Necroptosis

**Figure 7:**
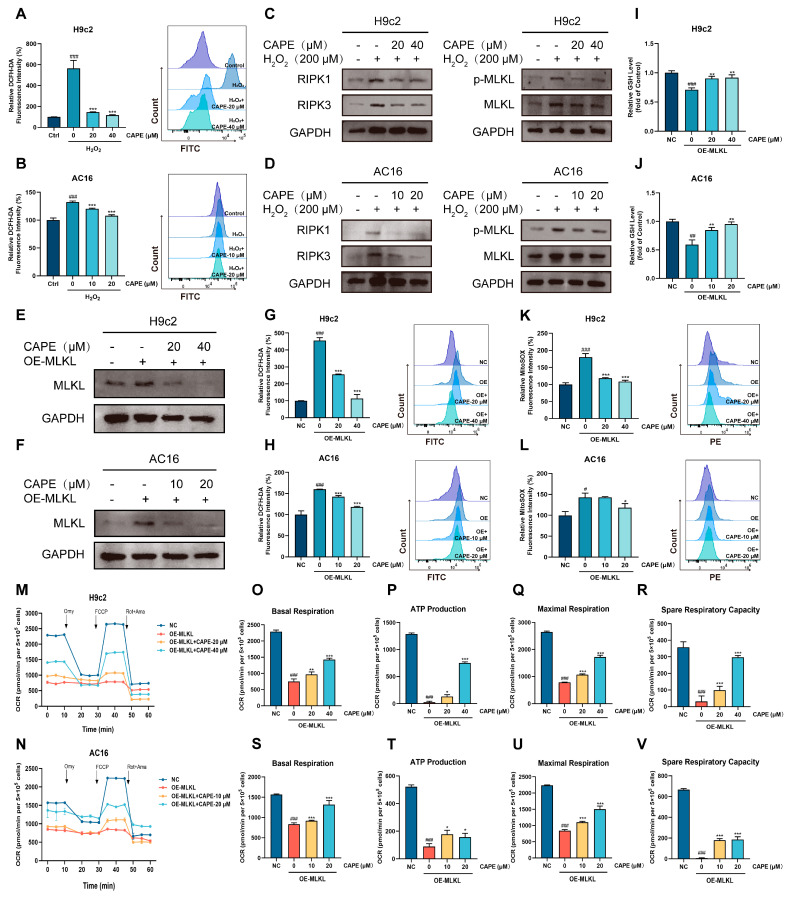
CAPE regulated the cross-talk mediated by the ROS-MLKL signal pathway. The H9c2 and AC16 cells were pre-treated with CAPE for 6 h, followed by 6 h of incubation with H_2_O_2_ (400 μM). (**A**,**B**) The ROS level was assessed and quantified by flow cytometry after the DCFH-DA staining. (**C**,**D**) The protein levels related to the RIPK1/RIPK3/MLKL pathway were examined by Western blotting. The H9c2 and AC16 cells were pre-treated with CAPE for 6 h, followed by 48 h of interference with H_2_O_2_. The values are represented as the $\bar{x}$ ± SD. *^###^ p* < 0.001 vs. control; *** *p* < 0.001 vs. H_2_O_2_ treatment group. (**E**,**F**) The protein level of MLKL was examined by Western blotting. (**G**,**H**) The ROS level was assessed and quantified by flow cytometry after DCFH-DA staining. (**I**,**J**) The GSH level was assessed. (**K**,**L**) The Mito-ROS level was assessed and quantified by flow cytometry after MitoSOX Red staining. (**M**,**N**) The OCR was examined by O2K cell energy metabolism analysis system. The basal respiration, ATP production, maximal respiration and spare respiratory capacity of the H9c2 (**O**–**R**) and AC16 (**S**–**V**) cells were quantified. Values are represented as the $\bar{x}$ ± SD. *^#^ p* < 0.05, *^##^ p* < 0.01, *^###^ p* < 0.001 vs. NC; * *p* < 0.05, ** *p* < 0.01, *** *p* < 0.001 vs. OE-MLKL group.

The above results confirm that there was a cross-talk between oxidative stress and necroptosis in the DOX-treated cardiomyocytes. Thus, it was instructive to explore whether CAPE could regulate that cross-talk to protect against DIC. H_2_O_2_ is a usual ROS inducer. We used H_2_O_2_ to induce an oxidative stress model instead of DOX for further research. As the results display, after the H_2_O_2_ administration in the H9c2 and AC16 cells_,_ the level of ROS increased significantly, indicating that the oxidative stress models were established (Figure 7A,B). Moreover, the expressions of key proteins in necroptosis, such as RIPK1, RIPK3 and p-MLKL/MLKL, were upregulated (Figure 7C,D). Interestingly, CAPE could inhibit the increased protein levels (Figure 7C,D), showing that CAPE may reduce cellular oxidative stress by inhibiting the expression of proteins in necroptosis. The above results indicate that CAPE modulated the cross-talk by inhibiting the ROS-mediated necroptosis.

Activated MLKL serves as the key executor of necroptosis, and its upregulation or activation has been shown to play a role in neurological, respiratory and cardiovascular diseases, as well as various types of cancer [37]. We overexpressed MLKL in cardiomyocytes, equivalent to the role of DOX, to activate necroptosis. As shown in Figure 7E,F, the transfection of the overexpressed MLKL plasmid successfully increased the protein expressions of MLKL in the H9c2 and AC16 cells, indicating that the necroptosis models were established. Meanwhile, we found that the overexpressed MLKL resulted in the upregulation of ROS and downregulation of GSH in the H9c2 and AC16 cells (Figure 7G–J). However, after the CAPE administration, the ROS levels and GSH contents were ameliorative (Figure 7G–J). The analysis of the Mito-ROS levels show similar results: in the H9c2 and AC16 cells, the CAPE pre-treatment ameliorated the overexpressed MLKL-induced increase in the Mito-ROS levels (Figure 7K,L). Furthermore, compared with the overexpressed MLKL group, the improved mitochondrial respiratory dysfunctions were observed in the H9c2 and AC16 cells after the treatment with CAPE (Figure 7M–V). The above results indicate that CAPE regulated the cross-talk by inhibiting the MLKL-mediated oxidative stress.

## 4. Discussion

As a powerful anti-tumor agent, DOX is strongly hampered by cumulative and dose-dependent cardiotoxicity in clinical applications [2]. There is an urgent need to investigate the mechanisms of DIC, as well as apply adjuvant therapies for preventing or ameliorating DIC. The only cardio-protectant approved currently by the U.S. FDA against anthracycline cardiotoxicity is Dex [4]. However, it may induce secondary malignancies and myelosuppression [5,6]. The monomer components extracted from natural products are good candidates for developing new drugs. Plenty of evidence suggests that CAPE has a wide range of pharmacological activities, especially antioxidation activity, which supports its cardioprotective effect (Figure 1) [38,39,40,41,42]. Under this background, the development of combination therapies with cardioprotective properties and the establishment of novel drug administration strategies that balance efficacy and safety have emerged as a pivotal direction in current cancer pharmacology research.

To investigate the pharmacological activity of CAPE, we established both in vivo and in vitro DIC models. Based on clinical protocols, the recommended dose of Dex is 20–30 times the therapeutic dose of DOX, administered 30 min prior to DOX. Therefore, Dex (90 mg/kg) was selected as the positive control regimen in this study to evaluate the in vivo efficacy of CAPE against DIC [43]. Preliminary experiments confirmed that 3 mg/kg DOX (intraperitoneal injection) significantly induced cardiotoxicity, while CAPE (0–40 mg/kg) showed no apparent systemic toxicity in mice. Guided by our previous studies, a 1-week pre-treatment protocol was adopted to assess the cardioprotective effects against DIC [44,45]. The different administration timelines for Dex and CAPE were designed to account for their intrinsic toxicities. In vivo experiments demonstrated that DOX-treated mice exhibited significant reductions in body weight and heart rate (Figure 2). A histopathological analysis revealed myocardial fiber disarray and fragmentation, accompanied by markedly elevated levels of the cardiac injury biomarkers cTn-I, CK-MB and LDH, which confirmed severe DIC (Figure 2). Notably, CAPE combination groups significantly attenuated these pathological alterations, which demonstrated comparable or superior efficacy to Dex. In vitro, MTT assays were employed to evaluate the relative cell viability of DOX-treated cardiomyocytes. The results confirmed that CAPE alone exhibited no cytotoxicity and significantly inhibited DOX-induced reductions in cardiomyocyte viability, which was consistent with the in vivo findings (Figure 3). Therefore, CAPE is expected to be applied in the preventive treatments of DIC, but its specific role or mechanism remains to be clarified.

An oxidative stress-induced increased level of ROS is believed to be the vital mechanism of DIC [21]. In addition, mitochondria are the key sites of ROS generation and the main targets of DOX. The redox cycle of DOX mainly occurs in the mitochondrial respiratory chain, resulting in the production of superoxide anions and ROS, while directly inhibiting the mitochondrial function of cardiomyocytes [46]. Thus, identifying a prospective approach to seek for drugs capable of maintaining ROS balance is critical for cellular health. In this study, CAPE was found to downregulate the DOX-induced increases in ROS levels, reduce declines in the glutathione (GSH) levels and inhibit mitochondrial ROS release. Mitochondria serve as both the primary source of ROS and a major target of oxidative stress (Figure 4). Excessive ROS exerts negative feedback on mitochondria, elevating mitochondrial oxidative levels, further damaging mitochondrial structure and function, and creating a vicious cycle that leads to severe cardiomyocyte injury. The TEM observations revealed that CAPE significantly alleviated the DOX-induced mitochondrial structural damage (Figure 4). Additionally, mitochondria, as the cellular “powerhouses”, generate ATP via oxidative phosphorylation and the tricarboxylic acid (TCA) cycle. In metabolically active cardiomyocytes, mitochondrial respiratory dysfunction is indicative of cellular pathology. Thus, the mitochondrial respiration levels in the H9c2 and AC16 cells were assessed using O2K mitochondrial energy analyzer, and the results further confirm that CAPE attenuated the DOX-induced oxidative stress (Figure 4).

Current studies have reported that multiple regulated cell death pathways contribute to the pathogenesis of DIC, and these multifactorial mechanisms are not independent, which may overlap or cross-talk, thereby increasing the complexity of DIC [21]. In this research, screening with multiple inhibitors identified those with the most pronounced impacts on DIC, further suggesting that oxidative stress and necroptosis may play a predominant role in DIC (Figure 5). The RIPK1/RIPK3/MLKL pathway is considered to be the classic regulatory pathway of necroptosis. After activation by external stimulation, such as oxidative stress or inflammation, RIPK1 interacts with RIPK3 to form a necrosome, which activates MLKL phosphorylation and enables its translocation to the plasma membrane, leading to cell death [26,29]. Thus, whether the classical pathway is involved in DOX-induced necroptosis needs to be investigated. Our results show that the expression levels of RIPK1, RIPK3 and p-MLKL/MLKL were significantly increased in vitro and in vivo, but the upregulated expression levels were significantly decreased by CAPE and Nec-1 (Figure 5). Taken together, our observations suggested that the CAPE could inhibit the RIPK1/RIPK3/MLKL-dependent necroptosis of cells caused by DOX.

The relevance between ROS and necroptosis has been proposed. Zhang et al. discovered that an intramolecular disulfide bond could be formed by ROS-mediated modification of the cysteine residues of RIPK1, which activated RIPK1 and, in turn, recruited RIPK3 to form the necrosome [47]. In addition, a study by Lu et al. presented that GSK872 (RIPK3 inhibitor) and Nec-1 protect retinal ganglion cells by inhibiting the RIPK1/RIPK3/MLKL pathway by not only reducing necroptosis but also decreasing the generation of ROS [48]. Moreover, TNF-mediated ROS was repressed by Nec-1 and RIPK3 knockdown in the mouse fibrosarcoma L929 cells [49]. However, the cross-talk between oxidative stress and necroptosis has not been specifically reported in the investigation of DIC mechanisms. Therefore, we further investigated the specific interaction mechanisms between these two pathways. In this study, we first demonstrated the cross-talk between oxidative stress and necroptosis using a DOX-induced cardiomyocyte model. NAC was found to suppress the DOX-induced upregulation of RIPK1/RIPK3/MLKL pathway proteins, which reversely confirmed that DOX could activate the RIPK1/RIPK3/MLKL signaling pathway through excessive ROS accumulation, thereby exacerbating necroptosis (Figure 6). Similarly, Nec-1 inhibited the DOX-induced elevation of total oxidative levels, suppressed mitochondrial ROS production, and alleviated mitochondrial damage and dysfunction (Figure 6). The results reveal that DOX could promote ROS generation by activating necroptosis, which led to mitochondrial damage and established a vicious cycle. These findings suggest that the cross-talk between oxidative stress and necroptosis may serve as a critical mechanism underlying the pathological progression of DIC.

Subsequently, to investigate the regulatory mechanism of CAPE on the cross-talk between oxidative stress and necroptosis, we established a cross-talk model by using two distinct approaches. We applied the ROS inducer H_2_O_2_ to simulate oxidative stress conditions and transfected with an overexpression plasmid encoding MLKL, the executioner gene of necroptosis, to specifically activate necroptosis. Our results show that after the CAPE pre-treatment, the upregulation of RIPK1/RIPK3/MLKL expression that was promoted by H_2_O_2_ was significantly decreased, indicating CAPE could inhibit the ROS-mediated necroptosis (Figure 7). Correspondingly, the oxidative stress caused by the overexpression of MLKL was also alleviated by CAPE (Figure 7). The results indicate that CAPE could regulate the cross-talk between the oxidative stress and necroptosis via inhibiting the ROS-MLKL signal pathway.

The cross-talk between oxidative stress and necroptosis represents the core mechanism of DIC. CAPE could intervene in the cross-talk through the ROS-MLKL axis, highlighting its unique advantage of dual regulation. But this study still had some limitations. Upstream regulatory factors, such as MAPK and NF-κB, have been demonstrated to play roles in the mechanisms of DIC [50,51]. However, whether they serve as cooperative targets for CAPE in regulating the cross-talk requires further validation. Therefore, in subsequent research, we will integrate multi-omics technologies to identify the upstream targets of CAPE in intervening in the cross-talk, providing a theoretical basis for revealing the pathological mechanisms of DIC and developing CAPE-based targeted strategies.

Currently, our results focus on the protective effect of CAPE against DOX-induced acute cardiotoxicity. Existing research has confirmed that CAPE synergistically enhances the anti-breast cancer efficacy of DOX and significantly improves the tumor sensitivity to DOX treatment [17,52], which suggests that we should explore CAPE’s effects on the tumor micro-environment and chemotherapeutic sensitivity in future investigations, aiming to elucidate the specific mechanisms underlying its dual pharmacological effects of “reducing toxicity and enhancing efficacy”. Such research will better promote the pre-clinical transformation of CAPE.

Achieving a clinical translation also requires the systematic evaluation of CAPE’s pharmacokinetic characteristics and safety. Existing pharmacokinetic studies on the single-dose intravenous administration of CAPE within low-dose ranges (0–20 mg/kg) have established preliminary pharmacokinetic foundations for clinical transformation [53,54,55], yet significant limitations remain. These include a lack of long-term and repeated administration data, as well as toxicity assessments for non-intravenous administration routes. Furthermore, studies have reported that CAPE exhibits non-linear dose–effect pharmacokinetic characteristics, complicating the prediction of the relationship between administered doses and drug exposure levels [55]. This unpredictability may increase the risk of adverse drug reactions. In addition, a prior study indicates that differences in esterase expression levels lead to varying hydrolysis activities toward drugs across species [56]. Carboxylesterases are abundantly expressed in rats, causing CAPE to be easily hydrolyzed and resulting in a short half-life. In contrast to rodents, humans have lower levels of carboxylesterases, which means that experimental data from rats cannot be directly extrapolated to humans [55]. Therefore, we will further conduct in-depth studies on the long-term toxicity and pharmacokinetics of CAPE using clinical samples or human induced pluripotent stem cell-derived cardiomyocytes (hiPSC-CMs) to reflect the conditions of the human body in the follow-up research, which will provide a basis for the design of clinical trial dosages, and thus, enhance the clinical application value of CAPE.

## 5. Conclusions

In conclusion, our study revealed that CAPE has powerfully cardioprotective effects against DIC and implicates that “the cross-talk between oxidative stress and necroptosis” may serve as the main mechanistic pathway through which CAPE protects against DIC. CAPE is expected to be applied as a cardioprotective agent for serving as an adjuvant therapy. This research on the efficacy and mechanism of CAPE in DIC also laid a scientific foundation for the development of innovative drugs and health products of propolis.

## Data Availability

The original contributions presented in this study are included in the article. Further inquiries can be directed to the corresponding author.

## Abbreviations

DOX, Doxorubicin; CAPE, Caffeic acid phenethyl ester; DIC, DOX-induced cardiotoxicity; ROS, reactive oxygen species; Dex, Dexrazoxane; FDA, Food and Drug Administration; TNFR1, Tumor necrosis factor receptor 1; RIPK1, Receptor-interacting serine/threonine-protein kinase-1; RIPK3, Receptor-interacting serine/threonine-protein kinase-3; MLKL, Mixed-lineage kinase domain-like; cTn-I, Cardiac troponin-I; CK-MB, Creatine kinase-MB; LDH, Lactate dehydrogenase; H&E, Hematoxylin and eosin; MTT, 3-(4,5-dimethyl-2-thiazolyl)−2,5-diphenyl-2Htetrazolium bromide; DMSO, Dimethyl sulfoxide; H_2_O_2_, Hydrogen peroxide; SPF, specific pathogen-free; NAC, N-acetylcysteine; Nec-1, Necrostatin-1; ECG, electrocardiogram; TEM, transmission electron microscopy; DCFH-DA, Dichlorodihydrofluorescein diacetate; GSH, Glutathione; OCR, oxygen consumption rate; Omy, Oligomycin; FCCP, Carbonyl cyanide-4-(trifluoromethoxy) phenylhydrazone; Rot, Rotenone; Ama, Antimycin A; IHC, immunohistochemistry assay.
